# Imaging Transcranial Direct Current Stimulation (tDCS) with Positron Emission Tomography (PET)

**DOI:** 10.3390/brainsci10040236

**Published:** 2020-04-15

**Authors:** Thorsten Rudroff, Craig D. Workman, Alexandra C. Fietsam, Laura L. Boles Ponto

**Affiliations:** 1 Department of Health and Human Physiology, University of Iowa, Iowa City, IA 52242, USA; craig-workman@uiowa.edu (C.D.W.); alexandra-fietsam@uiowa.edu (A.C.F.); 2 Department of Neurology, University of Iowa Hospitals and Clinics, Iowa City, IA 52242, USA; 3 Department of Radiology, University of Iowa Hospitals and Clinics, Iowa City, IA 52242, USA; laura-ponto@uiowa.edu

**Keywords:** tDCS, positron emission tomography, radiotracers, cortical excitability

## Abstract

Transcranial direct current stimulation (tDCS) is a form of non-invasive neuromodulation that is increasingly being utilized to examine and modify several cognitive and motor functions. Although tDCS holds great potential, it is difficult to determine optimal treatment procedures to accommodate configurations, the complex shapes, and dramatic conductivity differences among various tissues. Furthermore, recent demonstrations showed that up to 75% of the tDCS current applied to rodents and human cadavers was shunted by the scalp, subcutaneous tissue, and muscle, bringing the effects of tDCS on the cortex into question. Consequently, it is essential to combine tDCS with human neuroimaging to complement animal and cadaver studies and clarify if and how tDCS can affect neural function. One viable approach is positron emission tomography (PET) imaging. PET has unique potential for examining the effects of tDCS within the central nervous system *in vivo*, including cerebral metabolism, neuroreceptor occupancy, and neurotransmitter activity/binding. The focus of this review is the emerging role of PET and potential PET radiotracers for studying tDCS-induced functional changes in the human brain.

## 1. Introduction

Transcranial direct current stimulation (tDCS) is a form of non-invasive neuromodulation that is increasingly being applied to examine and modify several cognitive and motor functions, ranging from motor learning and working memory to performance fatigability and muscle strength [1,2,3,4]. tDCS devices typically consist of an adjustable direct current stimulator and two stimulating electrodes: a positive electrode (anode) and a negative electrode (cathode). These electrodes are placed over two separate locations on the scalp and a weak current, usually between 0.5–2.0 mA, flows between the electrodes and induces polarity specific shifts in cortical excitability [5]. Specifically, anodal stimulation increases cortical excitability and cathodal stimulation leads to excitability decreases, as measured with transcranial magnetic stimulation (TMS) [5]. However, this polarity-dependent change in excitability may be an oversimplification and the effects of tDCS are likely much more complicated [6]. There are several TMS-evoked motor outcomes that have been used to explore the modulatory effects of tDCS. For example, the amplitude of motor evoked potentials (MEP) are reflective of, and can be modulated by, spinal motor neuron excitability [6]. Accordingly, when applying a TMS pulse at a given intensity, any increase in MEP amplitude is thought to reflect an increase in synaptic excitability at the motor cortex or the spinal cord [7]. However, a review by Horvath et al. [8] questioned the efficacy of tDCS and suggested that MEP amplitude might be the only TMS outcome that is reliably modulated by stimulation. This was in contrast to other TMS measures derived from similar neural mechanisms (e.g., short interval intracortical inhibition (SICI), intracortical facilitation (ICF), and cortical silent period (cSP)) that were unaltered by tDCS [8]. On the other hand, the methods and conclusions of this review [8] have received sharp criticism by some tDCS experts [9]. Still, Vöröslakos et al. [10] also demonstrated that up to 75% of the tDCS current applied to rodents and human cadavers was shunted by the scalp, subcutaneous tissue, and muscle, further questioning the cortical effects of tDCS. Nevertheless, others [11] have shown preliminary evidence of little to no shunting from electrodes placed at the subthalamic level, which suggest that tDCS in rodents and human cadavers might not reflect the same stimulation properties as the living human. Therefore, the conflicting findings of [8,9] and [10,11] indicate that the effects of tDCS on the human brain is controversial and requires futher investigation via diverse methodological tools.

### tDCS Models and Simulations

Although tDCS has great potential, it is difficult to determine optimal treatment procedures to accommodate configurations, the complex shapes, and dramatic conductivity differences among various tissues (e.g., the scalp, skull, cerebrospinal fluid (CSF), gray matter, etc.). Usually, placing the stimulating electrodes directly over the targeted brain areas cannot guarantee the proposed modulation (excitation or inhibition) of those areas; although this is typically assumed when designing stimulation protocols. Computer simulations and tDCS modelling studies have helped identify the behavior of induced electrical currents over recent years [12]. While these models can be useful, they have many limitations. For example, they make assumptions about the conductivity of the underlying tissues, but different values can lead to highly variable results in electrical field magnitudes [13,14]. Other factors that might alter the electrical field include registration procedure errors, anatomic variations [15], functional connectivity, and inter-individual variability (e.g., age, gender, hormones, neurotransmitter levels, neuroanatomy). Furthermore, current distributions from animal studies do not translate well to human brains and postmortem electrical stimulation attempts are limited by differences between living and dead tissues [10]. This concept is also supported by the study of deceased patients with deep brain stimulation implants [16,17], in which neither the electric current paths nor their effects on living tissues were directly validated.

Consequently, it is essential to combine tDCS with human neuroimaging to complement animal and cadaver studies, and further clarify if and how tDCS can affect neural function. The physiological changes associated with tDCS in humans can be assessed with a variety of neuroimaging techniques. However, to-date the field has been led by non-invasive neuroelectrical techniques (EEG, MEG) and structural/functional magnetic resonance imaging (sMRI/fMRI). One viable alternative approach is positron emission tomography (PET) imaging. PET has unique potential for examining the effects of tDCS within the central nervous system *in vivo*, including imaging cerebral metabolism (e.g., glucose metabolism, oxygen utilization), neuroreceptor occupancy, and neurotransmitter activity/binding. Additionally, because PET imaging can provide a comprehensive (e.g., whole brain) image, it is ideally suited to not only investigate the effects of tDCS in areas directly under the stimulation electrodes but also in remote or functionally connected brain areas. 

**Figure 1:**
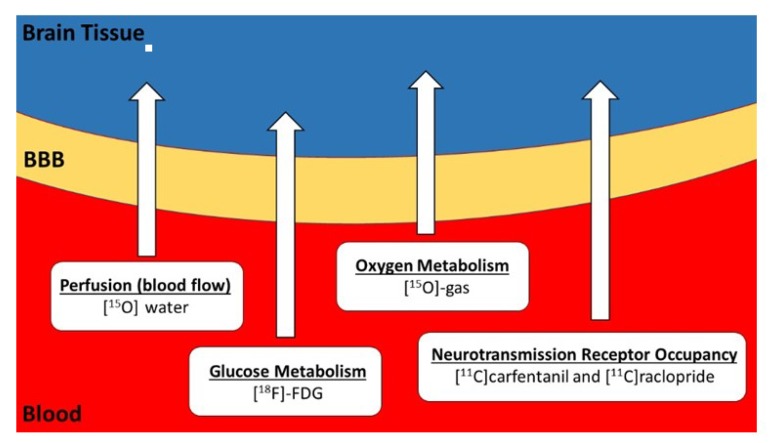
Potential PET tracers available for combination with tDCS. Glucose is the most important energy resource of the human brain and its metabolism can be measured and quantified using positron emission tomography (PET) with [^18^F]-fluorodeoxyglucose (FDG). Oxygen is necessary for the operation of the tricarboxylic acid cycle to synthesize ATP molecules from glucose, and oxygen metabolism can be measured using PET with [^15^O]-oxygen gas during inhalation. Glucose and oxygen molecules are supplied by blood flow. Brain regions with increased activity are accompanied by regional capillary dilation and increased regional cerebral blood flow (rCBF), which can be measured with [^15^O]water PET. The interaction of neurotransmitters and receptors can also be measured using PET with various [^11^C]-labeled ligands, such as [^11^C]carfentanil and [^11^C]raclopride. BBB = blood brain barrier.

However, as revealed in this review, PET-tDCS studies in humans are surprisingly sparse. Despite some inherent disadvantages of PET, in particular radiation exposure and high costs that limit large-scale application, many human tDCS research questions can be addressed exclusively with PET. PET is a functional imaging modality that characterizes a given functional process (e.g., metabolism, receptor density and binding, pathological burden (amyloid, tau)) contingent on the radiopharmaceutical employed. The most commonly used PET radiotracer is [^18^F]fluoro-deoxyglucose (FDG), a glucose analogue. Since the brain almost exclusively uses glucose as a fuel source, mapping FDG uptake provides insight into the distribution of brain activity. In addition, since the brain does not have nutrient stores, the delivery of glucose and oxygen, and the removal of waste products via cerebral blood flow (CBF), is tightly coupled to cerebral metabolism. The brain is extremely metabolically active and “activation” or “deactivation” of a particular brain region necessitates respective increases or decreases in metabolism of that area, accompanied by corresponding changes in CBF. FDG provides a means of mapping glucose metabolism, whereas, [^15^O]water, a freely-diffusible PET radiotracer, provides a means of assessing changes in CBF. The “metabolic trapping” properties of FDG and diffusivity of [^15^O]water PET tracers allows for the examination of the neuronal mechanisms underlying various tDCS applications in unconstrained conditions (i.e., under realistic training scenarios outside the scanner environment). Furthermore, the interaction of neurotransmitters and receptors can also be measured using PET with various [^11^C]-labeled (e.g., [^11^C]carfentanil and [^11^C]raclopride) or [^18^F]-labeled ligands. Figure 1 shows potential PET tracers that can be used to investigate the neural effects of tDCS.

The focus of this review is the emerging role of PET in the study of tDCS-induced functional changes in the human brain. Thus far, only a few studies have been published on this topic. This scarcity of research is difficult to comprehend, especially considering that neurological PET bears a unique potential for quantifying cerebral metabolism, blood flow, and receptor binding *in vivo* [18]. The current paper provides a contemporary overview of the available neurophysiological and neuroimaging data where tDCS has been used in combination with PET.

## 2. Measurements of Glucose Metabolism in tDCS Applications

PET imaging with the glucose analog, FDG, can quantify the uptake and metabolism of cerebral glucose, which is the primary substrate used in the genesis of ATP in the brain. Measuring FDG uptake in the CNS provides an estimate of brain activity [19,20] and is directly proportional to regional neuronal activity [21]. Because FDG is trapped in the brain during an initial uptake phase (10–30 min post injection) it also allows for the quantification of multiple brain regions during free-living activities, such as walking, running, or driving a car [22,23,24]. 

Consequently, this radiotracer can be injected during diverse test conditions and the resulting changes in cerebral glucose metabolism are measured after a known temporal delay. Thus, FDG-PET principally allows the visualization of “brain metabolic signatures” associated with different forms of motor and cognitive activities. Importantly, these “metabolic trapping” properties make it possible to inject FDG in realistic physical activity settings outside the PET scanner.

**Figure 2:**
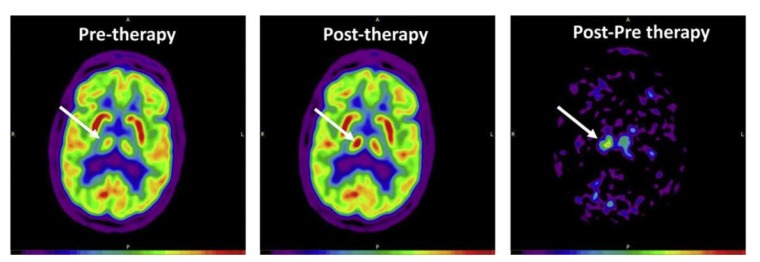
An example of FDG-PET image, taken from Rudroff et al. 2019 [25]. FDG-PET transaxial image acquired pre- and post-tDCS therapy. Images are scaled in standardized uptake values normalized to the global mean value (Max = 1.88 for pre-and post-therapy and 0.5 for post-pre therapy). The white arrow indicates the right thalamus, the area with the greatest difference between the images. The color bar describes increasing FDG uptake with increasing signal intensity (from black, indicating no glucose uptake, to red, indicating the greatest glucose uptake).

FDG-PET in humans has been used to investigate the effects of tDCS in patients with neuropathic pain [25,26], Alzheimer’s disease [27], healthy subjects [28,29], a patient with psychogenic non-epileptic seizures (PNES) [30], and patients in minimally conscious state (MCS) and unresponsive wakefulness syndrome (UWS) [31,32] (Table 1). These studies applied tDCS over the motor cortex (M1) or the dorsolateral prefrontal cortex (DLFPC) for 20–30 min with stimulation intensities ≤ 2 mA. Experimental designs with multiple sessions demonstrated improvements in pain ratings [25,26], cognitive function in Alzheimer’s Disease [27], online gaming addiction and self-control [29], and post-traumatic stress disorder (PTSD) symptoms, dissociative symptoms, depression, and alexithymia in a patient with PNES [30]. Importantly, FDG-PET provided valuable information about the underlying neural mechanisms of the described improvements, specifically alterations in the thalamus [25] (Figure 2), medulla [23], DLPFC [23,26], and the left middle/inferior temporal gyrus [27]. Furthermore, Zhang and colleagues [32] demonstrated that some residual brain activity was necessary to elicit a behavioral response to tDCS in MCS patients. Lee et al. [29] showed in young subjects (21 ± 1 years), that multiple tDCS sessions over the DLFPC resulted in improvements in online game addiction accompanied by less abnormal asymmetry of regional cerebral metabolic rate of glucose (rCMRglu) in the DLPFC. On the contrary, a single session of tDCS over the DLFPC resulted in no effects in patients with UWS [30]. Finally, Kraus et al. [28] conducted a tDCS intensity dose-response study (0.5 mA, 1 mA, and 2 mA for 10 min) with FDG-PET. Interestingly, they found no immediate changes in glucose consumption from any of the stimulation intensities.

### How tDCS Studies can Benefit from FDG-PET

Quantification of cerebral glucose metabolism allows researchers to determine the metabolic effects of tDCS interventions in humans and to relate regional brain metabolism with neuropsychological, behavioral, and physiological outcomes. Furthermore, FDG-PET under resting conditions has a strong promise for unraveling the influence of multiple tDCS sessions on neuronal integrity and for revealing potentially neuroprotective and restorative tDCS mechanisms *in vivo*, which is of particular interest in aging and neurological disease research. A major advantage of FDG-PET over other neuroimaging techniques, like fMRI, is that whole-body scans of a single subject cannot only provide information about the brain (Table 1), but also spinal cord activity [33], and skeletal muscle energy use [34,35]. Accordingly, future studies should investigate the interactions between energy use in the CNS and skeletal muscles after tDCS (e.g., in motor performance interventions). The studies summarized in Table 1 indicate that by using the “metabolic trapping” properties of FDG-PET, more detailed insights into the mechanisms of diverse tDCS applications (e.g., different intensities, durations, electrode montages, or brain targets) are likely to be gained. Moreover, the possibility of correlating glucose metabolism and FDG uptake with parameters of neurophysiological and psychological effects bears immense potential.

## 3. Measurements of Cerebral Blood Flow in tDCS Applications

The definitive gold standard for cerebral blood flow (CBF) determination is the injection of microscopic spheres, called microspheres, that travel in the blood and deposit into the capillaries. The deposition of the microspheres in a tissue is proportional to the blood flow (BF) that serves that tissue, and an understanding of the arterial input function and the concentration of deposited microspheres provides an accurate measure of tissue BF. However, microspheres cannot be used in humans because they permanently block the capillaries and determining their concentration is best done by “slice and dice” (i.e., sacrificing the animal and removing the tissue). The next best option for measuring CBF *in vivo* is [^15^O]water, which is a freely diffusible substance—i.e., it washes in and washes out in proportion to tissue BF (based on concentration gradients). If the arterial input function is known, the flow in the tissue can be converted to BF units (mL/min/100mL) by a convolution association, thus making it possible to look at the “real material” that is being moved between the tissues. [^15^O]water concentration is linearly proportional to BF to the tissue at lower flows and non-linear at higher flows (>65 mL/min/100mL); thus, [^15^O]water concentration at higher flows will underestimate actual BF. Nevertheless, higher flow is still associated with higher concentrations of [^15^O]water in the tissues. However, when an arterial line is not available, the regional CBF (rCBF) can be reported relative to a normalization value, like the BF of another brain structure (e.g., the cerebellum or thalamus) or the mean global CBF (gCBF). With the latter, the rate of BF is normalized relative to the average CBF of the whole brain and assumes that gCBF remains consistent even when rCBF might be altered. Because [^15^O]water PET is based on the use of the short-lived positron-emitting radionuclide oxygen-15 (half-life: 122 s), this radionuclide is ideal for measuring rCBF during cognitive or motor tasks that might be modulated by tDCS but is limited in its availability due to the need for a cyclotron to be in the immediate proximity of the PET scanner.

[^15^O]water PET imaging was validated as a measure of CBF in the early 1980’s by Raichle et al. [36] and Herscovitch et al. [37]. Since then, [^15^O]water PET has been considered the “gold standard” neuroimaging technique for measuring CBF in humans and was the imaging method of choice for the major, multi-center carotid artery occlusion study (COSS; [38]). [^15^O]water PET also serves as the validation method for newer CBF measurements, like arterial spin labeling (ASL: [39,40,41,42,43], multi-contrast MRI with deep convolutional neural network [44], and diffuse correlation spectroscopy [45]. Despite the great potential of [^15^O]water PET for investigating the immediate underlying mechanisms of tDCS, only three studies were identified for this review (Table 2). An early study by Lang et al. [46] combined M1 tDCS and [^15^O]water PET to clarify whether changes in rCBF were dependent on the functional state of the motor system (rest vs. movement) and tDCS polarity (anodal vs. cathodal). Both anodal and cathodal tDCS resulted in diffuse increases and decreases in rCBF in cortical and subcortical areas compared to sham. The magnitude of rCBF changes from tDCS were similar to the task-related changes during finger movements and were unchanged during the 50-min PET scanning time. Paquette et al. [47] showed that ΔrCBF of the M1 on the cathodal side was significantly lower than the anodal side M1 from active tDCS compared to sham. The cathodal decrease in ΔrCBF was also accompanied by depressed MEP amplitudes. These two studies applied 1 mA and 2 mA tDCS for brief durations (10 and 4 min), respectively. 

**Figure 3:**
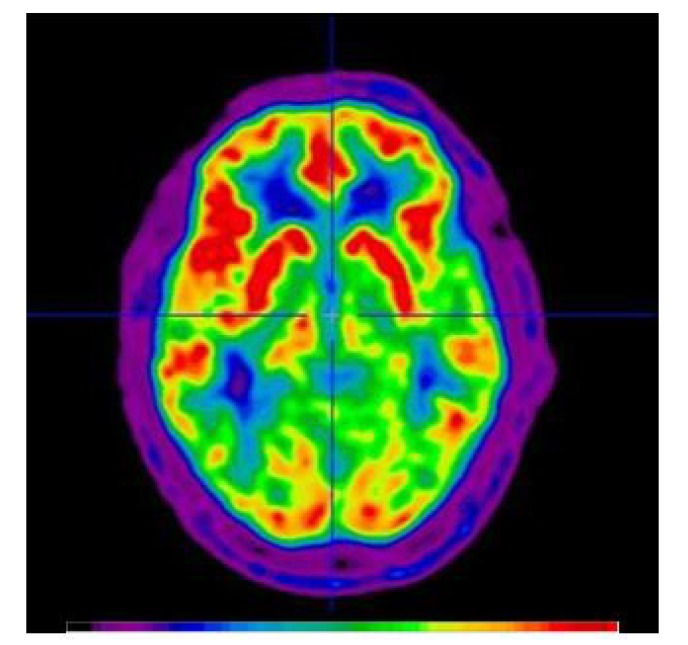
An example of [^15^O]Water Positron Emission Tomography (PET). The image is scaled in activity units (kBq/cc). The image was created by summing the first 40 seconds immediately post-bolus transit from a dynamic imaging sequence initiated at the time of [^15^O]water injection. The image is a semi-quantitative representation of the cerebral blood flow (CBF) at the time of bolus arrival in the brain. The color bar describes increasing blood flow with increasing signal intensity (from black to red).

However, the purpose of a very recent study by Workman et al. [48] was to investigate the effects of 5 min of DLFPC tDCS at different intensities (1 mA, 2 mA, 3 mA, and 4 mA) on CBF in people with multiple sclerosis. Their results revealed no immediate changes in rCBF from the different tDCS intensities. An example of a [^15^O]water PET image is presented in Figure 3.

### How tDCS Studies can Benefit from [^15^O]Water PET

[^15^O]Water is an ideal tracer to measure tissue BF because it is freely diffusible (chemically), has a short half-life, and can be combined with other tracers. In addition, metabolism and blood perfusion are closely associated, with the oxidative phosphorylation of glucose as a leading energy source. Thus, CBF is considered an indirect measure of neuronal function and integrity [49] and glucose metabolism is significantly correlated with different rCBFs across the brain and changes in gCBF in varying states of consciousness. Although many CBF studies use fMRI, there is great potential for tDCS studies to use [^15^O]water PET because this technique is considered the most “direct” measurement of CBF. The fMRI signal is often assumed to approximate the degree of local neural activation integrated over a spatial extent of several millimeters; however, this concept can be an oversimplification of the neurovascular coupling underlying many imaging sequences [50]. Furthermore, the combination of tDCS and fMRI has been technically challenging, requiring correction algorithms to adjust for image artifacts, and also has the remote risk of sudden, and potentially dangerous, electrode heating [51]; however, newer tDCS technologies are certified as safe for MRI scanning. On the other hand, tDCS in PET imaging presents fewer complications and tDCS-related imaging artifacts (e.g., from the electrodes or metal wires) can be minimized with computed tomography (CT) attenuation correction images, similar to correcting for metal fillings in the teeth. 

Finally, it is noteworthy that, to-date, no studies have combined [^15^O]O_2_ (oxygen) gas with tDCS. [^15^O]O_2_ gas can be safely administered via inhalation and provides detailed information about tissue oxygen consumption and extraction from the blood. This radionuclide could provide valuable tDCS mechanistic information and future studies could use [^15^O]O_2_ gas, either alone or in combination with [^15^O]water, to investigate the neuronal effects of tDCS. 

## 4. [^11^C]Carfentanil and [^11^C]Raclopride PET

**Figure 4:**
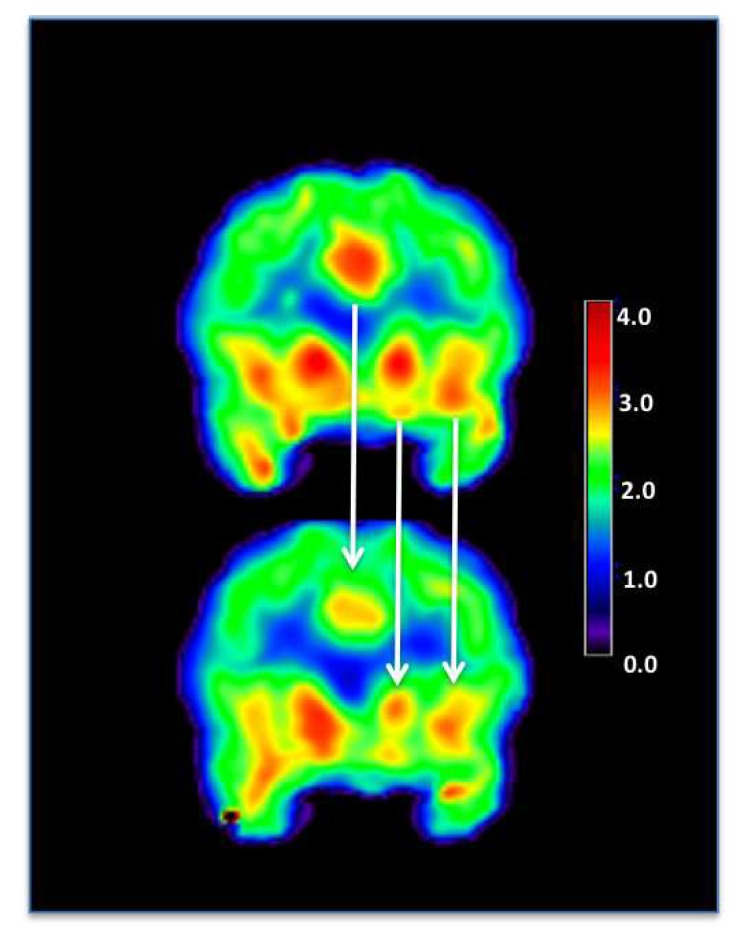
An example of [^11^C]carfentanil-PET image, taken from Dos Santos et al. 2012 [53]. Decrease in µ-opioid receptor (µ OR) binding associated with transcranial direct current stimulation. Upper panel: µ OR BPND during the baseline PET. Lower panel: µ OR BPND during active tDCS. ACC = anterior cingulate cortex; NAc = nucleus accumbens; Ins = insula; BPND = non-displaceable binding potential. The color bar describes increasing (µ OR) binding increasing signal intensity (from black to red).

PET receptor imaging can provide insight into the effects of interventions, such as tDCS, on neuroreceptor systems. Specific agents are designed to evaluate particular receptor systems. For example, two PET radiotracers ([^11^C]carfentanil and [^11^C]raclopride) have extended our knowledge of molecular mechanisms of pain management and cognitive function in the brain (Table 3). [^11^C]carfentanil is a strong and selective µ-opioid receptor (MOR) agonist. The µ-opioid system is the most important mechanism involved in the regulation of nociceptive signals and is a specific target of several opioid analgesics currently available for clinical use. Dos Santos et al. [52] used [^11^C]carfentanil to study the immediate effects of tDCS on MOR-mediated neurotransmission and heat-related pain thresholds in healthy subjects. Interestingly, they found that sham tDCS resulted in a decrease in MOR non-displaceable binding potential (BP_ND_) in the periaqueductal gray matter (PAG), precuneus, and thalamus, which indicates activation of the endogenous µ-opioid system. Active tDCS (2 mA for 20 min) also prompted MOR activation in the PAG and precuneus, but additionally increased MOR activation in the left prefrontal cortex. In another study, Dos Santos et al. [53] found that, despite the absence of clinical pain improvements in a subject with trigeminal neuropathic pain from post-herpetic neuralgia, one tDCS session significantly decreased MOR BP_ND_ levels in key pain-matrix structures, including the nucleus accumbens, anterior cingulate cortex, insula, and posterior thalamus (Figure 4). 

Dopamine plays an important role in many cognitive activities such as emotion regulation [54], reward-related processes [55], and executive functions [56] via the meso-cortico-limbic pathway. The effects of tDCS on this pathway can be investigated by PET with dopaminergic D2 subtype receptor availability via [^11^C]raclopride binding.

Fonteneau et al. [57] showed that a single session of tDCS over the DLFPC of both brain hemispheres (i.e., a bilateral/bihemispheric montage) induced dopamine release in cognitive and affective striatal areas. They further concluded that dopamine activity and reactivity levels might provide novel targets for brain modulation via bilateral tDCS over the DLPFC. Another group [58] used the same tracer to investigate cognitive changes in healthy human subjects after the dopamine system was activated by tDCS. They showed that tDCS over the DLPFC resulted in increased accuracy on a neuropsychological attentiveness test, which was significantly correlated with dopamine release in the right ventral striatum. Interestingly, the four above studies all showed significant effects on cerebral function after only a single application of 2 mA tDCS applied over M1 or DLFPC.

### How tDCS Studies can Benefit from [^11^C]Carfentanil and [^11^C]Raclopride PET

The [^11^C]carfentanil PET studies by Dos Santos et al. [52,53] present a more direct method for studying opioidergic mechanisms in tDCS research. Opioid receptor ligands exist with non-specific ([^11^C]Diprenorphine, [^18^F]Diprenorphine) or subtype-specific ([^11^C]carfentanil: μ-opioid receptor agonist; [^18^F]fluorocyclofoxy: μ/κ-opioid receptor antagonist) binding properties. Thus, it is now possible to image opioidergic receptor binding within the human CNS *in vivo* and to assess endorphin release-induced binding changes from tDCS. Future opioid and/or pain tDCS studies should therefore include these radionuclides to continue exploring the mechanisms of stimulation-related changes.

Dopamine is the principle neurotransmitter for central motor control and is involved in cognitive processing [59] and motivational behavior [60]. The two studies above [57,58] investigated the effects of tDCS via [^11^C]raclopride PET binding. These studies offer new insights for innovative uses of tDCS as a therapeutic aid for neuropsychiatric conditions involving dopamine transmission impairments, like the reward–motivation network, and suggest radiotracers for future tDCS work, particularly studies that target the DLPFC.

The above PET studies should be understood as ‘starting points’ that set the foundation for future ligand PET activation studies in tDCS. Future work may even focus on the interaction between the dopaminergic and opioidergic systems by using multi-tracer PET studies. It will also be possible to expand previous ligand studies using subtype specific opioidergic tracers and/or by determining extrastriatal dopaminergic binding changes using antagonistic tracers like [^18^F]fallypride [61], [^18^F]fluorodesmethoxy-fallypride [62], or [^11^C]FLB457 [63], all of which have been evaluated in humans to study extrastriatal dopamine D2/D3 receptors.

Furthermore, since PET provides insight into the dopaminergic system the complimentary use of magnetic resonance spectroscopy (MRS) allows for the quantification of neurochemicals, such as glutamate and γ-aminobutyric acid (GABA). MRS is becoming an increasingly useful tool in studying physiological changes induced to tDCS [64].

## 5. Discussion and Conclusions

tDCS is currently being promoted as an inexpensive and effective tool to enhance cognitive and behavioral function. However, the research exploring the efficacy of tDCS is far from conclusive. This review highlighted the potential role of PET as a molecular imaging technique for tDCS research. PET imaging provides distinct benefits and will be an important tool in the arsenal of different imaging techniques that seek to determine the mechanistic effects of tDCS. FDG-PET and [^15^O]water PET will serve as highly suitable biomarkers for testing whether tDCS counteracts cognitive impairments and motor dysfunction in the human brain. Additionally, receptor agents such as [^11^C]carfentanil and [^11^C]raclopride PET can be expected to play an important role in unraveling the neuroprotective and restorative effects of tDCS on the CNS. These latter radiotracers might have particularly interesting applications for refractory neurological symptoms (e.g., pain) and dopaminergic disorders. It should be noted, that PET studies are expensive and PET scanners are often less available to researchers. However, PET is a standard for cancer diagnosis and monitoring in most hospitals and clinics throughout the nation and additional usage for research is potentially viable worthwhile.

It can be concluded that PET with specific tracers, alone or in combination with other neuroimaging techniques, can provide further insights into the underlying mechanisms of tDCS. However, PET tDCS studies are currently in an exploratory stage and more research with larger sample sizes are needed.

Lastly, some critical points should not be left out. The risk/benefit ratio is different for treating and investigating symptoms with PET tDCS in diseased patients versus increasing motor and cognitive function in healthy subjects. While tDCS studies in clinical populations often require detailed risk assessments, the level of acceptable risk is different for healthy subjects and they might have a different risk/benefit ratio (e.g., more to lose and less to gain). PET uses radiotracers and subjects receive a dose of radiation during participation. For example, the amount of radiation from 15 mCi/dose of [^15^O]water is equivalent to approximately 25% of the annual natural background (environmental) radiation exposure and a 5 mCi/dose of [^18^F]-FDG is equivalent to approximately 11% of the annual radiation limit for a medical worker. These levels of radiation exposure are considered to be ethically appropriate for healthy subjects and routinely employed in research uses.

tDCS effects are highly variable among human subjects. Age [65,66], gender [66,67,68], hormones [69,70], handedness [71], cognitive ability [72], neurological or psychiatric disorders, medications [73], recreational drugs [74], neurotransmitter levels [75], prior exposure to brain stimulation [76], and differences in head anatomy [66,77] are important factors to consider because they have the potential to impact or even reverse a given tDCS effect. Regardless of all these uncertainties, the goal of most studies is to alleviate debilitating symptoms. Therefore, all of these potential sources of variation and risks/benefits should be taken into consideration when designing and conducting PET tDCS studies.

## Figures and Tables

**Table 1:** Summary of studies that investigated the effects of transcranial direct current stimulation (tDCS) with FDG-PET.

Study	Design	Subjects	Intervention	Main Findings
Rudroff et al. 2019 [25]	5-day intervention, case study	52-year-old man with multiple sclerosis-related neuropathic pain	Anode: left M1, Cathode: right supraorbital area 2 mA, 20 min	Pain scores improved after 5 sessions of tDCS. tDCS may induce functional changes in interconnected brain structures such as the thalamus.
Yoon et al. 2014 [26]	2 times/day for 10 days, double-blind	Patients with neuropathic pain, *N* = 16	Anode: left M1, Cathode: right supraorbital area, 2 mA or sham, 20 min	Significant decrease in the numeric rating scale scores for pain after tDCS. Increased metabolism in the medulla and decreased metabolism in the left DLPFC after active tDCS treatment.
Im et al. 2019 [27]	1 time/day for 6 months	Patients with early Alzheimer’s disease *N* = 18, sham = 7, tDCS = 11	Anode: left, DLPFC, Cathode: right DLPFC, 2 mA, 30 min	tDCS improved global cognition. rCMRglc in the left middle/inferior temporal gyrus was preserved in the active group, but was decreased in the sham group.
Kraus et al. 2019 [28]	Acute effects, sham-controlled, randomized, single-blind, crossover trail	Healthy subjects *N* = 15	Anode: left DLPFC, Cathode: right DLPFC, 0.5 mA, 1 mA, 2 mA, 10 min at each intensity	tDCS did not yield significant changes in glucose consumption at any tested stimulation intensity in this paradigm.
Lee et al. 2019 [29]	3 times/week for 4 weeks	Healthy subjects Online gamers, *N* = 15 Non-gamers, *N* = 10	Anode: left DLPFC, Cathode: right DLPFC, 2 mA, 30 min	tDCS sessions lowered the IAT score and weekly hours spent playing games, and improved BSCS scores. The abnormal asymmetry of rCMRglu in the DLPFC, where the right side was greater than the left side, was improved after the tDCS sessions in the gamer group.
Leroy et al. 2019 [30]	5 days/week, 2 times/day, for 3 weeks, case study	39-year-old woman with PNES	Anode: left DLPFC, Cathode: right DLPFC, 2 mA, 30 min	Hypometabolism of the anterior associative cortical areas, involving the bilateral dorsolateral prefrontal cortex and to a lesser extent the bilateral orbitofrontal cortex. Improvement in PTSD symptoms, dissociative symptoms, depression, and alexithymia.
Thibaut et al. 2015 [31]	Acute affects, sham-controlled, randomized, double-blind, crossover trial	Patients with sub-acute or chronic MCS *N* = 21	Anode: left DLPFC, Cathode: right supraorbital area 2 mA, 20 min	Hypometabolism in non-responders as compared with responders in the left DLPFC, the medial-prefrontal cortex, the precuneus, and the thalamus. EEG did not show any difference between the two groups.
Zhang et al. 2020 [32]	20 anodal tDCS sessions over 10 consecutive days (2 daily sessions), sham-controlled, randomized, double-blind	Patients with UWS (*N* = 13) or MCS (*N* = 5), and healthy controls (*N* = 6)	Anode: left DLPFC, Cathode: right DLPFC, 2 mA, 20 min	The residual brain metabolism in the left DLPFC in MCS patients supported that residual brain activity in the stimulated area was necessary for a behavioral response to tDCS.

Note: In all studies, FDG-PET was performed after tDCS. FDG = [^18^F]fluorodeoxyglucose; PET = positron emission tomography; M1 = primary motor cortex; DLPFC = dorsolateral prefrontal cortex; rCMRglc = regional cerebral metabolic rate of glucose; IAT = Internet Addiction Test; BSCS = Brief Self Control Scale; PNES = psychogenic non-epileptic seizures; PTSD = post-traumatic stress disorder; MCS = minimally conscious state; EEG = electroencephalography; UWS = unresponsive wakefulness syndrome.

**Table 2:** Summary of studies that investigated the effects of transcranial direct current stimulation (tDCS) with [^15^O]water PET.

Study	Design	Subjects	Intervention	Main Findings
Lang et al. 2005 [46]	Acute effects, sham-controlled, single-blind, crossover design	Healthy subjects *N* = 16	Anode: M1, Cathode: right frontopolar cortex, 1 mA, 10 min	Anodal and cathodal tDCS induced widespread increases and decreases in rCBF in cortical and subcortical areas. These changes in rCBF were of the same magnitude as task-related rCBF changes during finger movements and remained stable throughout the 50-min period of PET scanning.
Paquette et al. 2011 [47]	Acute effects, sham-controlled, single-blind	Healthy subjects *N* = 9	Anode: dominant M1, Cathode: non-dominant M1, 2 mA, 4 min	ΔrCBF of the M1 on the cathodal side was significantly lower than the anodal side M1 from active tDCS compared to sham. The cathodal side decrease in ΔrCBF was also accompanied by depressed MEP amplitudes.
Workman et al. 2020 [48]	Acute effects (single session), sham-controlled, single-blind, crossover design	Patients with multiple sclerosis *N* = 3	Anode: left DLPFC, Cathode: right supraorbital area, 1 mA, 2 mA, 3 mA, and 4 mA, 5 min each	No immediate changes in rCBF from 5 min of tDCS at 1 mA, 2 mA, 3 mA, and 4 mA.

Note: [^15^O]water PET was performed after tDCS [46] and during tDCS [47,48]. M1 = primary motor cortex; rCBF = regional cerebral blood flow; PET = positron emission tomography; MEP = motor-evoked potential; DLPFC = dorsolateral prefrontal cortex.

**Table 3:** Summary of studies that investigated the effects of transcranial direct current stimulation (tDCS) with [11C]carfentanil and [11C]raclopride PET.

Study	Design	Subjects	Intervention	Main Findings
Dos Santos et al. 2012 [53]	Acute effects (single session), sham-controlled, single-blind, case study	62-year-old woman with trigeminal neuropathic pain from post-herpetic neuralgia	Anode: primary M1, Cathode: supraorbital region, 2 mA, 20 min [^11^C]carfentanil PET	No changes in clinical pain improvements. Significantly decreased MOR BP_ND_ levels in key pain-matrix structures, including the nucleus accumbens, anterior cingulate cortex, insula, and posterior thalamus.
Dos Santos et al. 2014 [52]	Acute effects (single session), sham-controlled, single-blind	Healthy subjects *N* = 9	Anode: right M1, Cathode: left supraorbital region, 2 mA, 20 min [^11^C]carfentanil PET	Sham tDCS resulted in a decrease in MOR BP_ND_ in the periaqueductal gray matter (PAG), precuneus, and thalamus, which indicates activation of the endogenous µ-opioid system. Active tDCS (2 mA for 20 min) also prompted MOR activation in the PAG and precuneus, but additionally increased MOR activation in the left prefrontal cortex.
Fonteneau et al. 2018 [57]	Acute effects (single session), sham-controlled, double-blind	Healthy subjects *N* = 32, Sham = 18, tDCS = 14	Anode: left DLPFC, Cathode: right DLFPC, 2 mA, 20 min [^11^C]raclopride PET	A single session of bilateral DLFPC tDCS induced dopamine release in cognitive and affective striatal areas.
Fukai et al. 2019 [58]	Acute effects, sham-controlled, double-blind, crossover design	Healthy subjects *N* = 20	Anode: left DLPFC, Cathode: right DLPFC, 2 mA, 13 min × 2 [^11^C]raclopride PET	tDCS over the DLPFC resulted in increased accuracy on a neuropsychological attentiveness test, which was significantly correlated with dopamine release in the right ventral striatum.

Note: PET was performed during tDCS [47,48,52] and after tDCS [53]. M1 = primary motor cortex; MOR = µ-opioid receptor; BP_ND_ = non-displaceable binding potential; DLPFC = dorsolateral prefrontal cortex.
